# Improvement of occipital neuralgia by pulsed radiofrequency of the greater occipital nerve combined with local magnesium sulfate injection: a randomized controlled study

**DOI:** 10.3389/fphys.2026.1838823

**Published:** 2026-05-05

**Authors:** Jialei Zhang, Zhishan Zhang, Enquan Song, Jie Wu

**Affiliations:** Department of Pain Treatment, Changzhi People’s Hospital Affiliated to Changzhi Medical College, Changzhi, China

**Keywords:** greater occipital nerve, magnesium, occipital neuralgia, pain, pulsed radiofrequency

## Abstract

**Objective:**

To investigate the safety and efficacy of magnesium sulfate as an adjuvant in pulsed radiofrequency (PRF) stimulation of the greater occipital nerve for the treatment of occipital neuralgia.

**Methods:**

Consecutive patients diagnosed with neurogenic occipital neuralgia who attended the Pain Department of Changzhi People’s Hospital were recruited for this study, randomly assigned to control group and experimental group. The control group received PRF treatment on the affected side only, whereas the experimental group received an additional perineural injection of 5% magnesium sulfate (150 mg) around the greater occipital nerve immediately after PRF. NRS scores, pain duration, and pain frequency were compared between the two groups at baseline (T0), 30 days post-treatment (T1), and 60 days post-treatment (T2), at these identical time points, ultrasonography was performed to measure the thicknesses of the ipsilateral semispinalis capitis, obliquus capitis inferior, and deep fascia superficial to the obliquus capitis inferior, along with vertebral artery systolic flow parameters. Additionally, shear wave elastography (SWE) was used to measure the maximum shear wave velocity (SWVmax) and mean shear wave velocity (SWVmean) of the semispinalis capitis to assess cervical musculature status.

**Results:**

A total of 91 patients completed the entire study, with 43 in the control group and 48 in the experimental group. No significant between-group differences were observed preoperatively in occipital neuralgia symptoms (NRS scores, pain duration, and frequency), thickness of the ipsilateral perineural muscles and fascia, shear wave elastography of the semispinalis capitis, or vertebral artery hemodynamic parameters (*P*>0.05). Pain symptoms were significantly reduced after treatment compared with preoperative levels in both groups(*P* < 0.05), and patients in the experimental group exhibited significantly alleviated pain symptoms compared with the control group (*P* < 0.05). No significant differences were detected in muscle, fascia, or vertebral artery parameters in the control group between pre- and post-treatment assessments (*P*>0.05). In contrast, all parameters of muscle, fascia, and vertebral artery in the experimental group were significantly improved post-treatment compared with baseline (*P* < 0.05).

**Conclusion:**

Local magnesium-sulfate injection enhances pulsed radiofrequency efficacy by relieving perineural muscle spasm and improving vertebral-artery hemodynamics, leading to sustained reductions in headache intensity, duration, and frequency with an excellent safety profile.

## Introduction

Occipital neuralgia is a common chronic neuropathic pain disorder characterized by recurrent unilateral electric-shock or stabbing pain, often accompanied by scalp tenderness and paresthesias (Blake and Burstein, 2019). Its prevalence among patients with primary headache disorders is approximately 1.4–4.6%, with a slight female predominance (Chowdhury et al., 2021), and it markedly impairs quality of life and social function. The pathogenesis is primarily related to compression, injury, or inflammatory irritation of the greater and/or lesser occipital nerves (Swanson et al., 2022). Current evidence suggests that multiple factors contribute, including cervical muscle spasm, degenerative cervical spine changes, local inflammatory responses, and demyelinating alterations of the nerve (Attal et al., 2023).

At present, management of occipital neuralgia consists of acute symptomatic treatment and preventive therapy. Acute attacks are commonly treated with non-steroidal anti-inflammatory drugs (NSAIDs), triptans, or ergot alkaloids; however, these agents are ineffective in some patients, and prolonged use may lead to medication-overuse headache (MOH) and other adverse effects (Buse et al., 2019). Preventive options include β-blockers, antidepressants, and antiepileptic drugs, yet hepatorenal toxicity and additional side-effects limit their widespread application (Allam et al., 2023).

Pulsed Radiofrequency (PRF) technology is a minimally invasive technique that uses either thermal energy from RF current or pulsed electric fields to modulate or ablate neural tissue. In recent years it has been widely applied to neuropathic pain conditions such as trigeminal neuralgia and post-herpetic neuralgia, offering the advantages of minimal invasiveness, precision, high safety, and a marked reduction in post-operative pain recurrence (Wu et al., 2019). Ultrasound-guided RF ablation of the greater occipital nerve has proven to be an effective and safe option for occipital neuralgia (Orhurhu et al., 2021).

Magnesium sulfate is a commonly used adjuvant analgesic whose mechanism involves modulating neurotransmitter release (e.g., glutamate), inhibiting N-methyl-D-aspartate (NMDA) receptors, and regulating calcium influx (Avci et al., 2024). Clinically, magnesium sulfate has been shown to enhance the efficacy of local anesthetics and reduce post-operative analgesic consumption (de Oliveira Filho et al., 2023). In addition, it exerts anti-inflammatory effects by suppressing the nuclear factor-κB (NF-κB) pathway and decreasing the release of pro-inflammatory cytokines (Sehnert et al., 2020).

Although PRF ablation has achieved notable success in treating occipital neuralgia, studies on its combination with adjuvant agents remain limited. The use of magnesium sulfate as an adjunct during PRF procedures has not been adequately investigated. This randomized controlled trial was designed to evaluate the therapeutic effect of ultrasound-guided greater occipital nerve PRF ablation combined with magnesium sulfate injection in occipital neuralgia, aiming to provide a novel, effective treatment strategy that improves patient outcomes and quality of life.

## Materials and methods

### Study design

This study is a single-center, prospective, triple-blind, randomized controlled trial designed to evaluate the role of magnesium sulfate as an adjuvant when perineural injection is added to pulsed radiofrequency (PRF) of the greater occipital nerve for occipital neuralgia. Headache score, a continuous variable, was chosen as the primary efficacy endpoint. With a two-arm, parallel-group design, α (two-sided) = 0.05, power = 0.80 (β = 0.20), and an anticipated dropout rate of 10%, the sample-size formula indicated that at least 40 patients per group (≥ 80 in total) were required.

The study protocol was approved by the Medical Ethics Committee of Changzhi People’s Hospital affiliated to Changzhi Medical College and was registered with the Chinese Clinical Trial Registry (registration No. ChiCTR2500103747). Written informed consent was obtained from all participants.

### Patients

This study enrolled patients aged 40–70 years of either sex who presented to the Department of Pain Medicine, Changzhi People’s Hospital affiliated to Changzhi Medical College, between July and December 2025 for pulsed radiofrequency treatment of neurogenic occipital neuralgia. Inclusion criteria required fulfillment of the 2018 ICHD-3 diagnostic criteria for occipital neuralgia issued by the International Headache Society (Headache Classification Committee of the International Headache Society (IHS), 2018), disease duration exceeding three months, unilateral occipital pain, at least 50% reduction in headache after a diagnostic greater occipital nerve block, inadequate response to pharmacotherapy (the patients received analgesia solely via oral anti-inflammatory analgesics, which showed no significant improvement in pain scores or pain frequency), no prior pulsed radiofrequency procedure within the preceding three months, good compliance allowing completion of all study procedures, and non-participation in any other clinical trial. Exclusion criteria comprised history of cervical spine trauma, bleeding disorders, epileptic or other seizure disorders, neurological disease, severe hepatic or renal impairment, uncontrolled diabetes mellitus, pregnancy or lactation, and refusal to participate.

### Randomization, allocation concealment and blinding

One investigator obtained written consent and collected baseline data during the pre-operative visit. A second investigator used SPSS to randomly assign participants to either the control group or the perineural magnesium-sulfate group (experiment group), sealed the assignment in an opaque envelope, and clipped it into the patient’s chart. After the patient entered the operating room, a third investigator opened the envelope in an anesthesia preparation area remote from the operating theater, prepared the study syringe according to the allocation, and handed it to the proceduralist for perineural injection immediately after the radiofrequency lesion. Post-discharge follow-up and outcome data collection were performed by the first investigator, who remained unaware of group assignment throughout.

### Procedure

After the patient entered the operating room, standard monitoring (ECG, non-invasive blood pressure, and pulse oximetry) was applied and an intravenous line was established. The patient was placed in the lateral decubitus position with the symptomatic side uppermost and asked to relax the cervical musculature. A 5 MHz convex ultrasound probe (Mindray M6, China) was positioned between the C1 transverse process and the C2 spinous process to obtain a clear view of the greater occipital nerve and the posterior cervical muscles and fascia. A cannula was then inserted under real-time ultrasound guidance. Once satisfactory needle placement was confirmed, the cannula was connected to a radiofrequency generator (Beiqi, Beijing). Motor (0.5–2 mA, 2 Hz) and sensory (0.5–1 mA, 50 Hz) stimulation tests were performed to verify correct positioning, after which pulsed radiofrequency treatment was delivered at 42 °C, 2 Hz, 45V for 200s. Immediately after PRF completion, the experimental group received 3 mL of 5% magnesium sulfate (150 mg) injected perineurally between the semispinalis capitis and obliquus capitis inferior muscles, whereas the control group received 3 mL of 0.9% saline at the same site.

### Data collection and outcome measures

Baseline data: age, sex, body-mass index, and comorbidities.

Headache intensity: The numeric rating scale (NRS) was used to evaluate headache intensity scores at baseline (T0), 30 days (T1), and 60 days (T2) post-treatment, with comparisons made between the two groups.

Headache duration: Maximum duration of single headache episode (hours) — baseline and post-treatment observation period.

Headache frequency: episodes per week documented for the same two intervals.

Assessment of perineural muscular and fascial thickness: At T0, T1, and T2 time points, a diagnostic ultrasound machine (Mindray M6, China) equipped with a 5 MHz convex ultrasound probe was used, with the probe was positioned at the C1 transverse process and C2 spinous process to obtain a standard plane clearly visualizing the posterior cervical muscles and fascia. The thicknesses of the ipsilateral semispinalis capitis, obliquus capitis inferior, and deep fascia superficial to the obliquus capitis inferior were measured at 1.50 cm lateral to the C2 spinous process. Each measurement was repeated three times, and the average was calculated.

**Figure 1:**
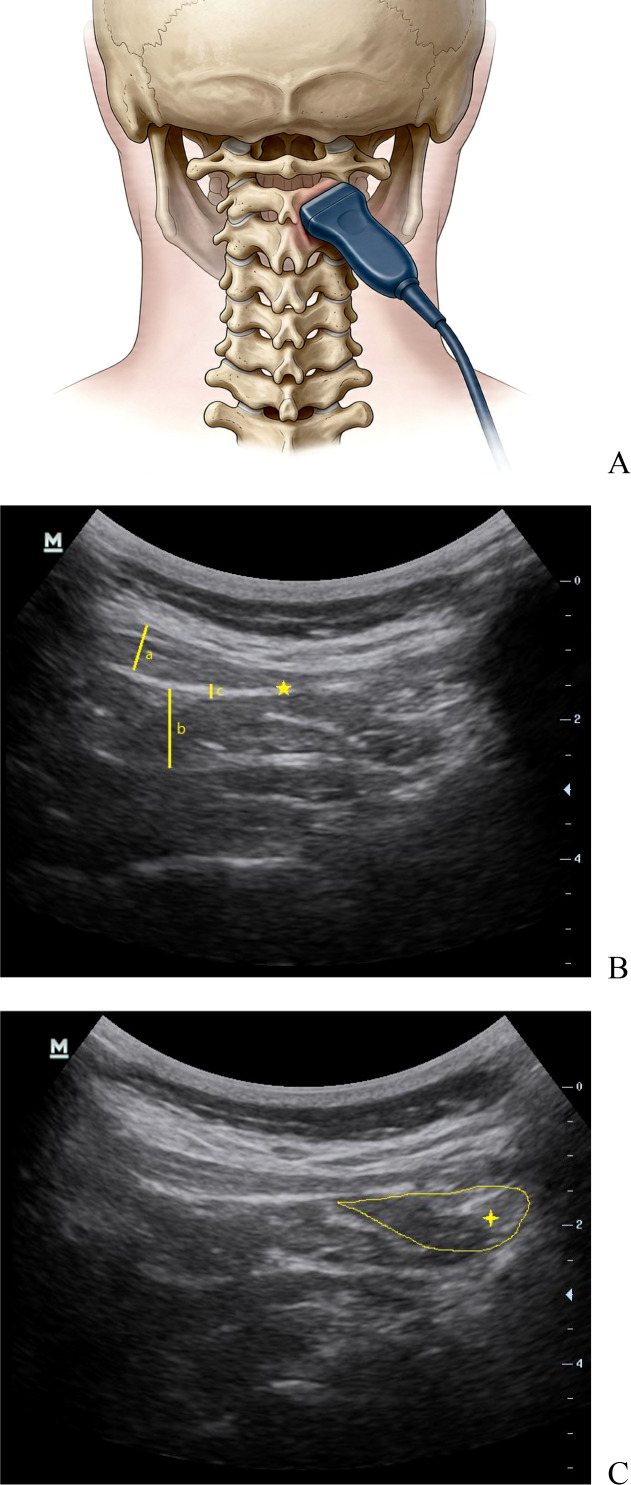
Sonographic visualization of the greater occipital nerve and perineural muscular structures. **(A)** Ultrasound probe placement: the probe was uniformly placed between the C1 transverse process and C2 spinous process. **(B)** Transverse sonogram between the C1 transverse process and C2 spinous process: the greater occipital nerve is located superficial to the obliquus capitis inferior muscle and deep to the semispinalis capitis muscle. (a) semispinalis capitis thickness (SCT) (B) obliquus capitis inferior thickness(OCIT) (c) the deep fascia superficial to the obliquus capitis inferior thickness (DF-SOCI) ★: the greater occipital nerve **(C)** Imaging of drug distribution following injection ✦: drug distribution following injection.

In SWE mode, the sample box was set to a uniform size and placed along the long axis of the semispinalis capitis, maximum shear wave velocity (SWVmax) and mean shear wave velocity (SWVmean) of the region of interest were measured three times and averaged as the final result. (**Figure 1**).

**Figure 2:**
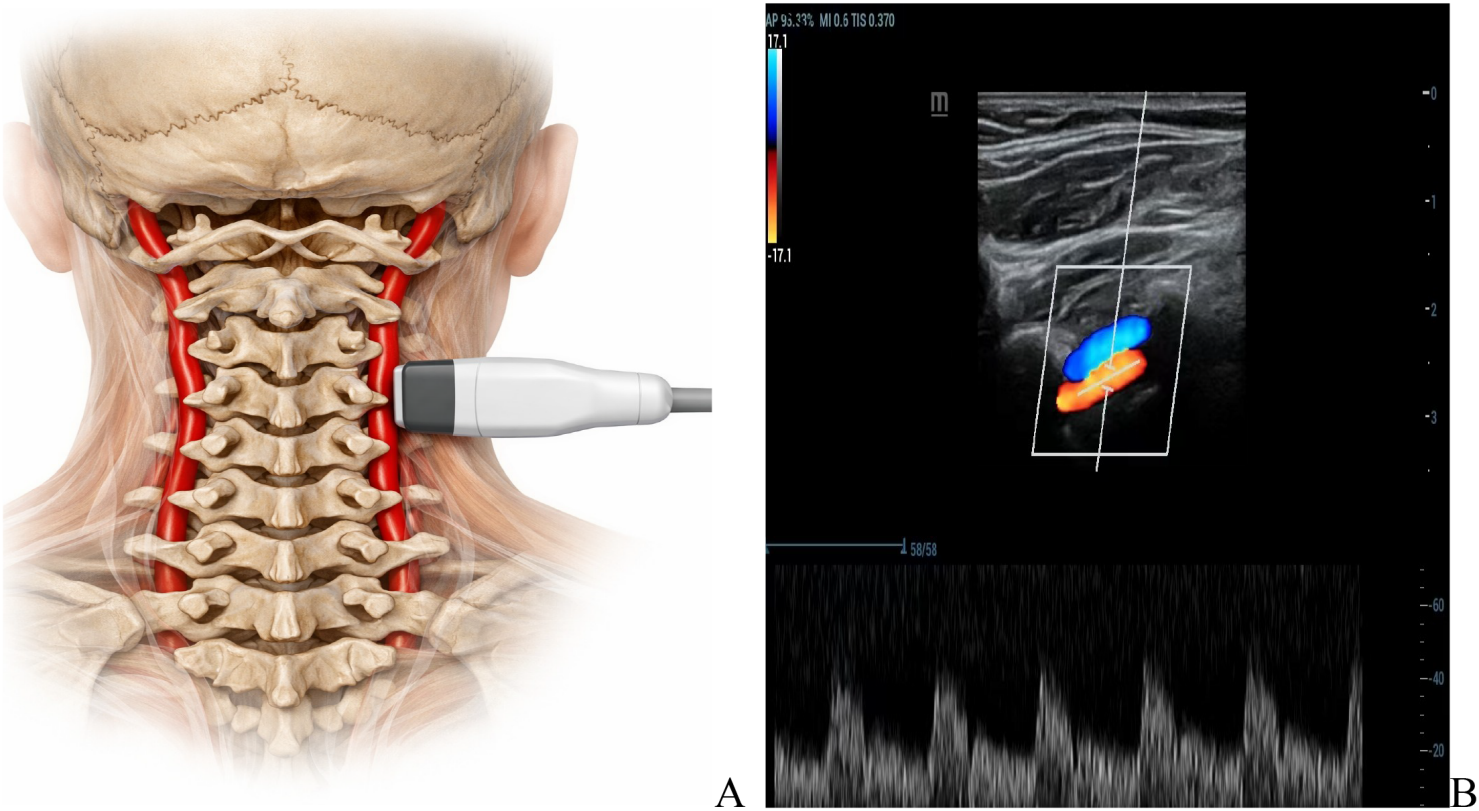
Ultrasound monitoring of vertebral artery parameters. **(A)** Probe placement for ultrasound monitoring of vertebral artery parameters. The transducer was positioned at the level of the C4-C6 transverse processes to visualize the vertebral artery through the intertransverse space. **(B)** Ultrasonic evaluation of vertebral artery hemodynamics including flow velocity, directional patterns, pulsatility index, and spectral Doppler waveform characteristics.

Vertebral-artery hemodynamics: At T0, T1, and T2 time points, A 10 MHz linear ultrasound probe (Mindray Resona 7, China) was used to examine the hemodynamics of the ipsilateral vertebral artery through the sub-occipital window. For each examination, the same depth was maintained as consistently as possible on the same arterial side, and hemodynamic parameters including systolic velocity (VS), pulsatility index (PI), and resistance index (RI) were recorded. (**Figure 2**).

Rescue analgesia: After admission, patients were allowed to continue their routine analgesic medications prior to treatment, but all analgesics were discontinued from the day of treatment. If post-treatment NRS≥4, acetaminophen 300 mg was administered. The number of patients requiring rescue medication and the total number of doses per group were recorded and compared between groups.

### Statistical analysis

All data were analyzed with SPSS 26.0. Continuous variables are presented as mean ± SD when normally distributed and as median (inter-quartile range) otherwise. Between-group comparisons of means were performed with the independent-samples t test; if normality was violated, the Mann–Whitney U test was used. Within-group changes were assessed with the paired t test; when the paired differences were not normally distributed, the Wilcoxon signed-rank test was applied. Categorical variables are reported as frequencies or percentages and compared with the *χ*² test. A two-tailed *P* < 0.05 was considered statistically significant.

## Results

### Comparison of general conditions

**Figure 3:**
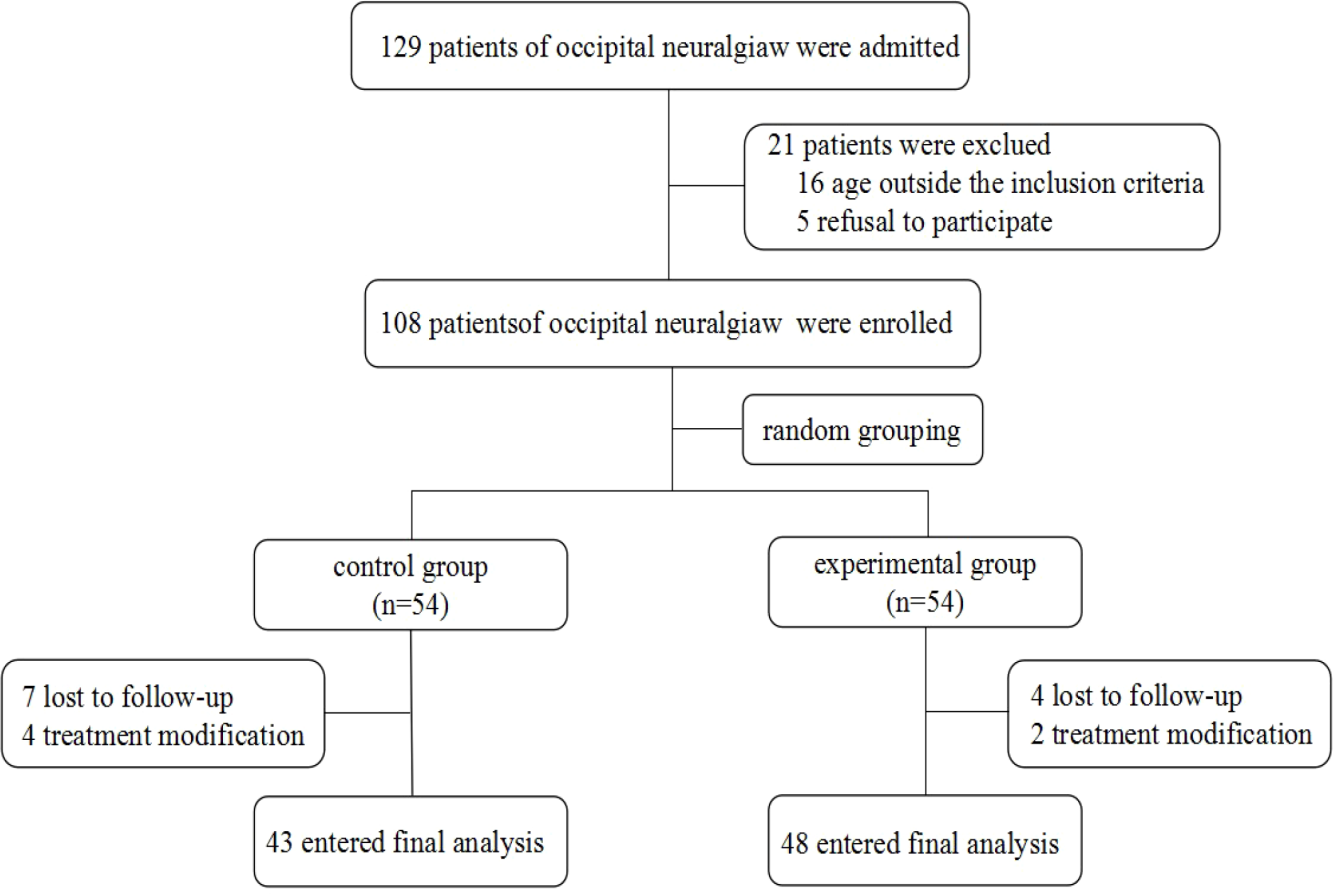
Flow chart of the study.

From July to December 2025, the Pain Department of Changzhi People’s Hospital Affiliated to Changzhi Medical College admitted a total of 129 patients with occipital neuralgia; 108 met the inclusion criteria, and ultimately, were enrolled and completed the entire trial, 43 in the control group and 48 in the experimental group. The two groups showed no significant differences in sex, age, comorbidities, smoking history, and alcohol consumption history (*P*>0.05). (**Figure 3**, **Table 1**).

**Table 1:** Demographic and baseline characteristics.

Variable	Control group (n=43)	Experimental group (n=48)	*P* value
Age, year	57.49 ± 7.31	56.52 ± 8.38	0.561
Sex, n (%)			
Male	16 (37.2)	20 (41.7)	0.414
Female	27 (62.8)	28 (58.3)	
BMI, kg/m2	23.66 ± 2.89	24.39 ± 2.88	0.231
Chronic smoking, n (%)	19 (44.2)	17 (35.4)	0.393
Alcoholism, n (%)	6 (14.0)	8 (16.7)	0.793
History of surgery, n (%)	14 (32.6)	13 (27.1)	0.568
Comorbidity, n (%)			
Hypertension	21 (48.8)	27 (56.3)	0.479
Diabetes mellitus	15 (34.9)	19 (39.6)	0.644
Coronary heart disease	3 (7.0)	5 (10.4)	0.563
Chronic lung disease	2 (4.7)	1 (2.1)	0.493

### Comparison of pain parameters before and after treatment between two groups

Preoperative comparisons revealed no significant between-group differences in NRS scores, headache duration, or headache frequency (all *P*>0.05).

Following treatment, repeated measures ANOVA showed significant time effects for all parameters (*P* < 0.001). *Post-hoc* Bonferroni tests revealed significant reductions in the experimental group at both T1 and T2 compared with T0 (all *P* < 0.001), and similarly in the control group (all *P* < 0.001).

Between-group comparisons demonstrated significantly lower NRS scores, shorter headache duration, and reduced headache frequency in the experimental group versus control group at T1 (*P* < 0.05) and T2 (*P* < 0.05), suggesting markedly greater headache relief in the experimental group.

**Figure 4:**
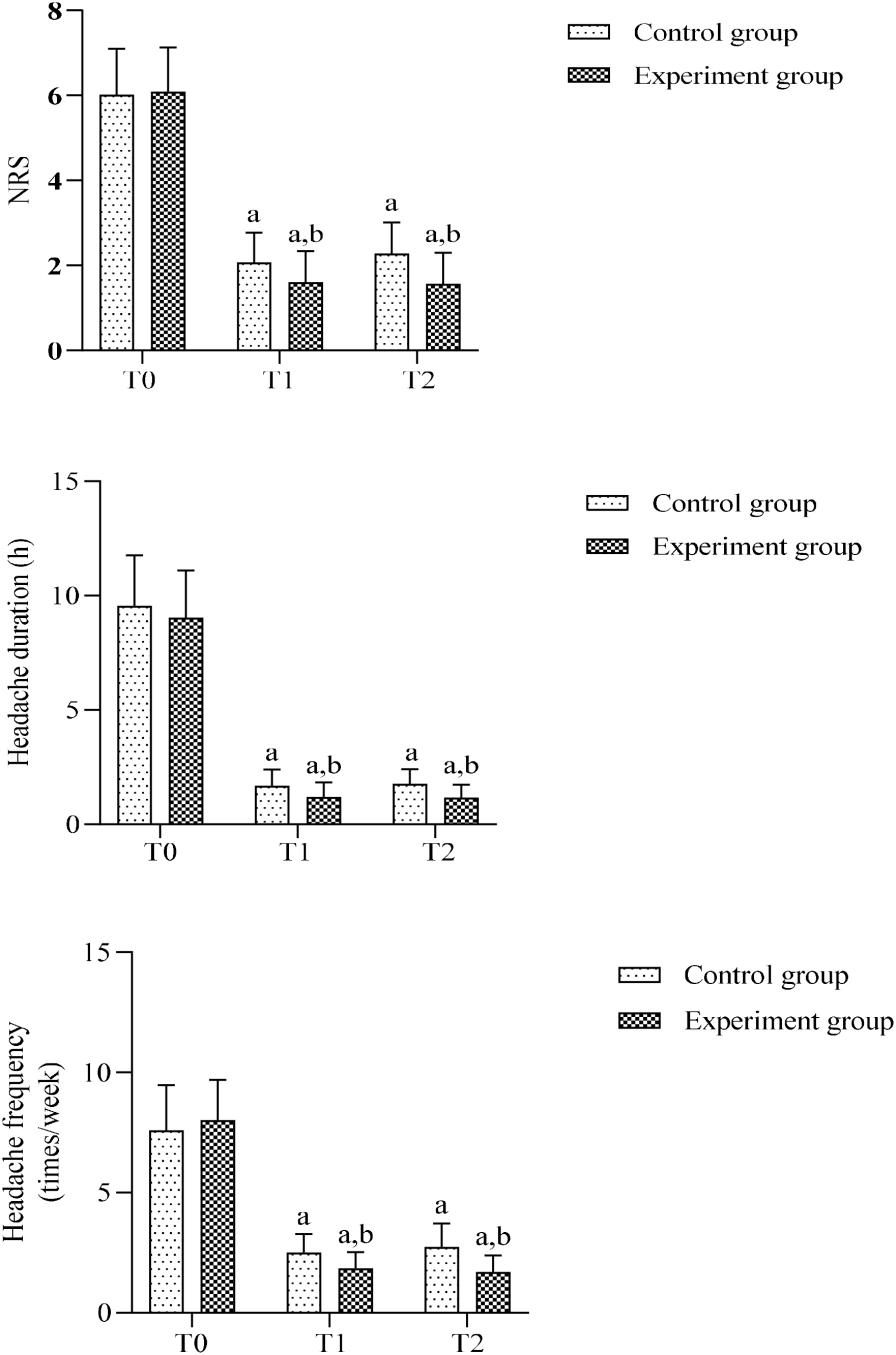
Comparison of pain parameters before and after treatment between two groups. (a) Intra-group comparison with T0, *P* < 0.05; (b) Inter-group comparison at the same time point, *P* < 0.05.

Intragroup comparisons between T1 and T2 showed no significant differences in either group (all *P*>0.05), indicating sustained treatment effects, indicating sustained treatment effects. (**Figure 4**, **Table 2**).

**Table 2:** Comparison of NRS scores, headache duration, and headache frequency between groups.

Groups	NRS			Headache duration (h)			Headache frequency (times/week)		
	T0	T1	T2	T0	T1	T2	T0	T1	T2
Control group (n=43)	6.02 ± 1.08	2.07 ± 0.70^a^	2.28 ± 0.73^a^	9.56 ± 2.21	1.69 ± 0.71^a^	1.77 ± 0.65^a^	7.60 ± 1.87	2.51 ± 0.77^a^	2.74 ± 0.98^a^
Experimental group (n=48)	6.06 ± 1.04	1.60 ± 0.74^a^	1.56 ± 0.74^a^	9.12 ± 2.06	1.19 ± 0.64^a^	1.17 ± 0.56^a^	8.02 ± 1.68	1.85 ± 0.68^a^	1.71 ± 0.68^a^
*t*	0.177	3.075	4.625	0.968	3.607	4.746	1.115	4.321	5.906
*P*	0.860	0.003	<.0.001	0.335	0.001	< 0.001	0.268	< 0.001	< 0.001

^a^Intra-group comparison with T0, *P* < 0.05.

### Comparison of perineural muscle and fascial thickness between the two groups

**Figure 5:**
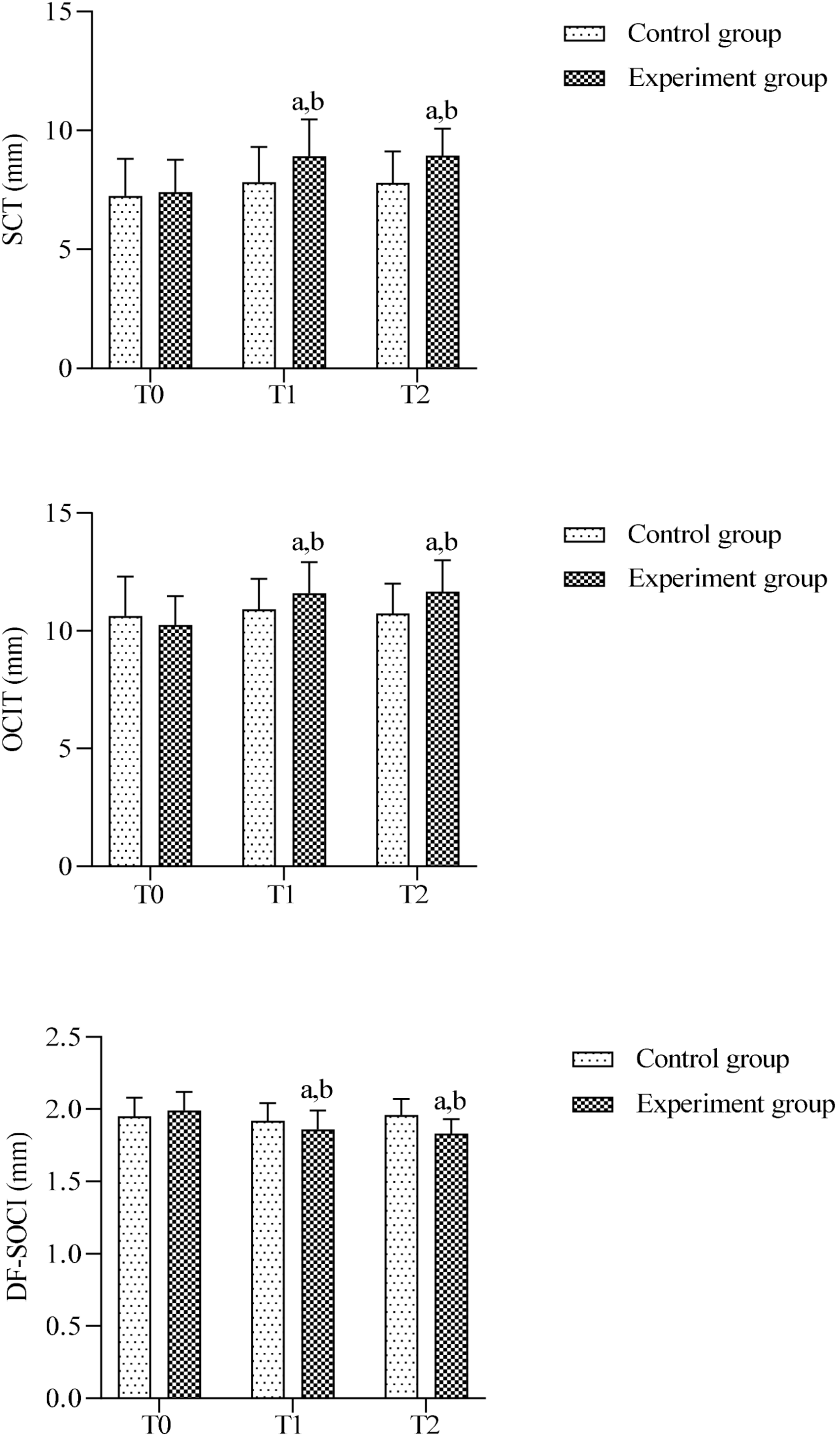
Comparison of muscle and fascia thicknesses between groups. SCT, semispinalis capitis thickness; OCIT, obliquus capitis inferior thickness; DF-SOCI, thickness of deep fascia superficial to obliquus capitis inferior. (a) Intra-group comparison with T0, *P* < 0.05; (b) Inter-group comparison at the same time point, *P* < 0.05.

Before treatment, no significant differences were observed between the two groups in the semispinalis capitis thickness(SCT), obliquus capitis inferior thickness(OCIT), or the deep fascia superficial to the obliquus capitis inferior thickness(DF-SOCI) (*P*>0.05). After treatment, the control group showed no significant changes in any of these parameters compared with baseline (*P*>0.05), whereas the experimental group exhibited a significant increase in the thickness of both the semispinalis capitis and obliquus capitis inferior (*P* < 0.05) and a significant decrease in the thickness of the deep fascia superficial to the obliquus capitis inferior (*P* < 0.05). Furthermore, inter-group comparisons at each post-treatment time point revealed that the experimental group had significantly greater thickness of the semispinalis capitis and obliquus capitis inferior (*P* < 0.05) and significantly reduced thickness of the deep fascia superficial to the obliquus capitis inferior compared with the control group (*P* < 0.05). (**Figure 5**).

### Comparison of shear-wave elastography of the semispinalis capitis between the two groups

**Figure 6:**
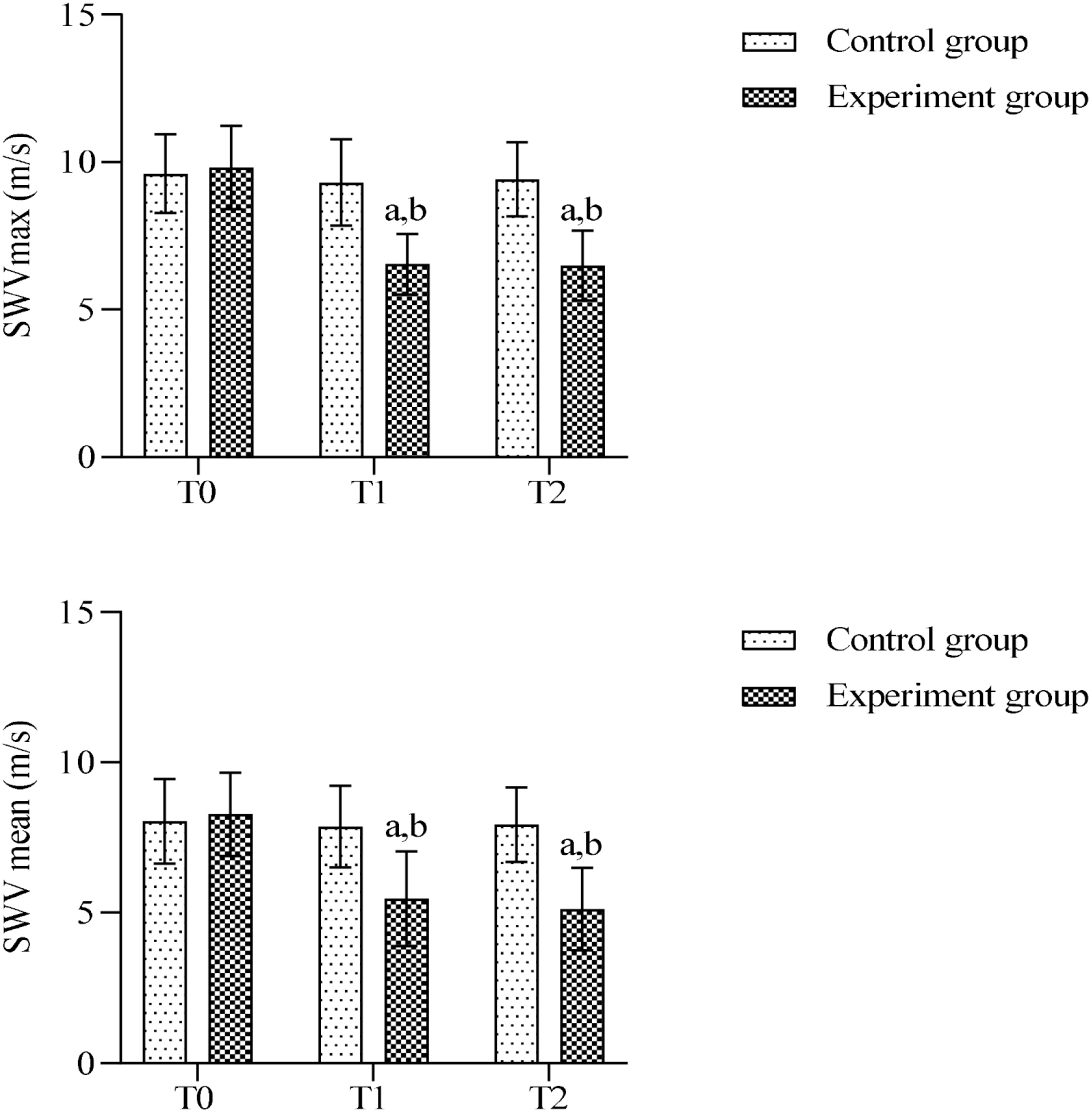
Comparison of SWVmax and SWVmean between groups. SWVmax, Maximum shear-wave velocity; SWVmean, Mean shear-wave velocity (a) Intra-group comparison with T0, *P* < 0.05; (b) Inter-group comparison at the same time point, *P* < 0.05.

Shear-wave velocity (SWV) was measured in the ipsilateral semispinalis capitis of patients in both groups. At preoperative, SWVmax and SWVmean did not differ significantly between the groups (*P*>0.05). After treatment, no significant changes in SWVmax or SWVmean from baseline were observed in the control group at any postoperative time point (*P*>0.05). In the experimental group, both SWVmax and SWVmean were significantly decreased from baseline and compared with the control group at all post-treatment time points (*P* < 0.05), with no statistically significant difference observed between T1 and T2 (*P*>0.05). (**Figure 6**).

### Comparison of vertebral artery blood flow parameters on the affected side between two groups

**Figure 7:**
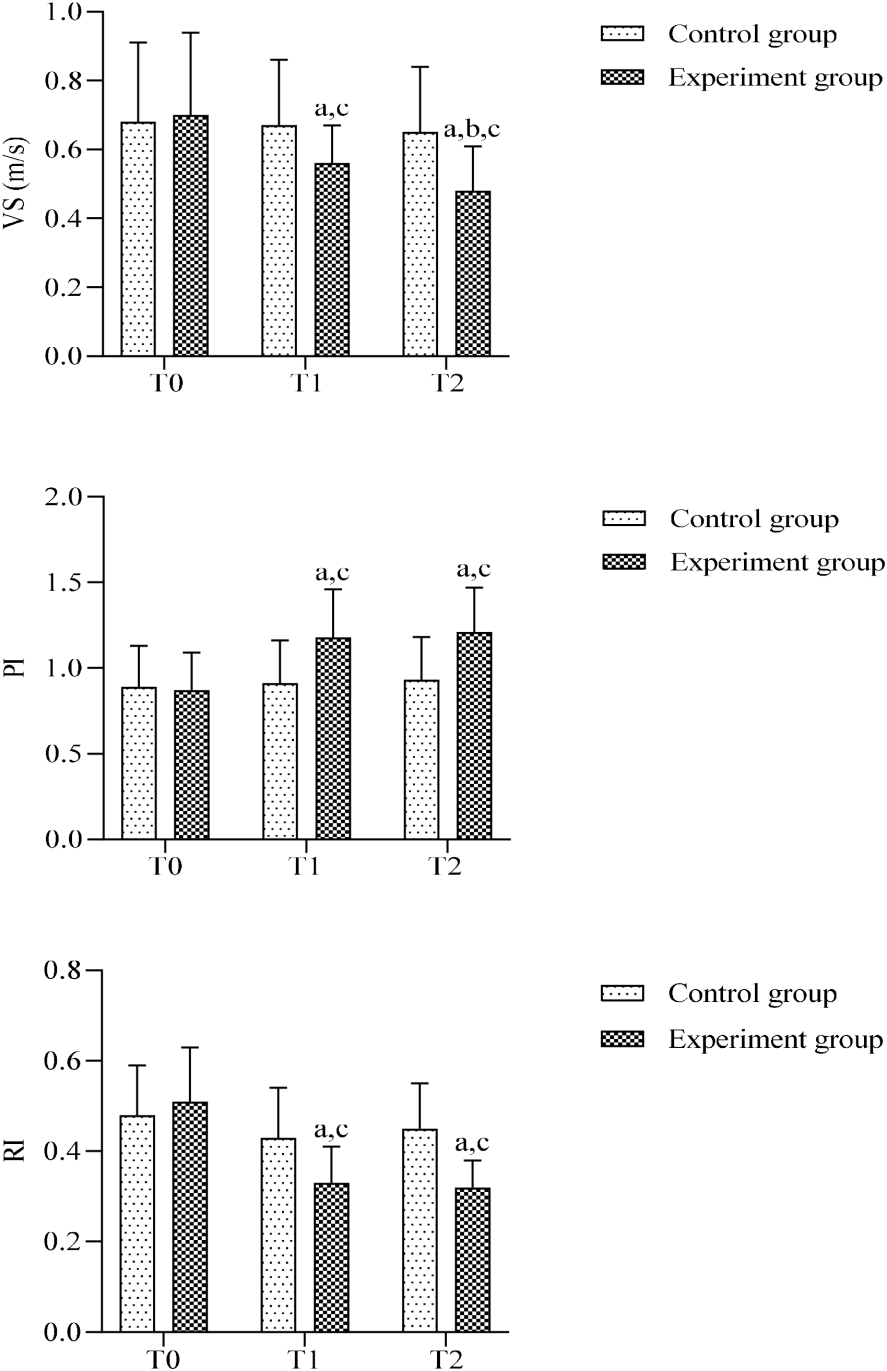
Comparison of affected vertebral artery hemodynamics between groups. VS, the peak velocity systolic; PI, pulsatility index; RI, resistivity index (a) Intra-group comparison with T0, *P* < 0.05; (b) Intra-group comparison with T1, *P* < 0.05; (c) Inter-group comparison at the same time point, *P* < 0.05.

Comparison of the ipsilateral vertebral artery systolic velocity (VS), pulsatility index (PI), and resistance index (RI) between the two groups before and after treatment showed no significant differences in any hemodynamic parameters at baseline (*P*>0.05). No significant changes in VS, PI, or RI were observed in the control group after treatment (*P*>0.05). In contrast, the experimental group exhibited significantly decreased VS and RI, and significantly increased PI compared with preoperative values (*P* < 0.05), all of which were significantly superior to the control group, indicating more favorable improvement of vertebral artery blood flow circulation in the experimental group after treatment. (**Figure 7**).

### Complications and supplemental analgesic use

No pulsed radiofrequency-related complications occurred in either group; intra- or post-operative nausea, vomiting, hypotension, or bradycardia were not observed. In the experimental group, no magnesium-sulfate–associated toxic effects—such as changes in respiratory rate, degree of muscle relaxation, or deep-tendon reflexes—were detected. Moreover, both the number of patients requiring supplemental analgesics and the total number of doses were significantly higher in the control group than in the experimental group (*P* < 0.05).

## Discussion

In previous studies, magnesium sulfate was used solely as an adjuvant to regional anesthesia, enhancing the analgesic effect of local anesthetics and prolonging the duration of analgesia (Zeng et al., 2021). However, other investigations have reported negative findings, concluding that magnesium sulfate neither potentiates local-anesthetic analgesia (Haghighi et al., 2015; Ramegowda et al., 2024) nor reduces postoperative opioid consumption (Bisui et al., 2017). These discrepancies may be attributable to differences in surgical procedure, timing and route of administration, dosage, and individual sensitivity to anesthetic agents. To eliminate such confounders, the present study employed magnesium sulfate alone for perineural injection and evaluated the feasibility and efficacy of this agent as an adjunct to pulsed radiofrequency therapy for occipital neuralgia. Our results demonstrate that both treatment modalities decrease pain intensity and frequency, yet the addition of perineural magnesium sulfate to pulsed radiofrequency further reduces post-treatment headache frequency, prolongs the therapeutic effect, and markedly improves vertebral artery blood flow.

After its origin from the posterior ramus of the second cervical nerve, the greater occipital nerve passes between the obliquus capitis inferior and semispinalis capitis, gradually ascends toward the superficial plane, pierces the deep cervical fascia, and reaches the subcutaneous tissue, where it divides into several sensory terminal branches that supply the skin above the superior nuchal line and the posterior scalp. Because the nerve traverses a complex, variable course through muscle, fascia, joints, and adipose tissue, it is readily irritated at any point along this pathway, leading to entrapment, inflammation, or chronic strain that produces episodic or continuous knife-like, electric-shock, or dull pain in its distribution (Kristoffersen et al., 2018). Pulsed radiofrequency delivers high-current bursts in a stepwise manner, raising tissue temperature to 42 °C, and exerts non-destructive neuromodulatory effects. Its mechanism involves an electromagnetic field that reduces substance P release and stimulates endogenous analgesic substances such as enkephalins, thereby achieving long-lasting analgesia through altered synaptic transmission, ion channel regulation (sodium and calcium channels), and suppression of inflammatory signaling pathways (ERK/MAPK) (Lin et al., 2025), and promotes partial neural repair (Jorge et al., 2022). This technique is now widely applied in various neuropathic pain conditions, including trigeminal neuralgia (Zhao et al., 2025), sciatica (Napoli et al., 2023), and post-herpetic neuralgia (Saxena et al., 2016).Our findings mirror these earlier reports. After pulsed radiofrequency treatment, occipital-neuralgia patients exhibited significant reductions in pain scores, attack frequency, and duration, confirming the marked therapeutic efficacy of pulsed radiofrequency for this condition.

The core pathogenesis of occipital neuralgia is a vicious cycle of cervical-muscle dysfunction and nerve entrapment. Prolonged poor posture, acute strain, or muscular imbalance induces tension, spasm, or aseptic inflammation in the muscles along the course of the greater occipital nerve, directly compressing or irritating the nerve as it emerges or tugging on the surrounding fascia, thereby evoking occipital radiation pain (Gfrerer et al., 2022). The resulting neurogenic reflex spasm further intensifies muscle contraction and entrapment, creating a self-perpetuating loop of “muscle tension--nerve compression--escalating pain--compensatory spasm” (Urits et al., 2020). Through myofascial-chain compensation this loop amplifies pain signals (Caballero Ruiz de la Hermosa et al., 2023), producing chronic or recurrent symptoms. Magnesium sulfate, a peripherally and centrally acting muscle relaxant, relieves spasm-related pain via multiple, well-defined ion-channel mechanisms. Mg^2+^ competitively blocks voltage-gated calcium channels, curbing acetylcholine release at the neuromuscular junction and lowering motor-end-plate excitability, thereby directly relaxing skeletal muscle (Shin et al., 2020). Simultaneously, Mg^2+^ functions as a physiological N-methyl-D-aspartate (NMDA)-receptor antagonist, preventing calcium influx and central sensitization and modulating descending pain pathways (Pinto et al., 2024). Recent research shows that magnesium sulfate also exerts anti-inflammatory effects by inhibiting cytokine cascades, a property especially relevant in neuropathic pain (Yue et al., 2022). Shear-wave elastography can quantify muscle mechanics; shear-wave velocity precisely reflects semispinalis capitis stiffness, allowing accurate assessment of muscular stress (Zhang et al., 2023). In the present study, adjunctive perineural magnesium sulfate added to pulsed radiofrequency significantly increased the thickness of the obliquus capitis inferior and semispinalis capitis, decreased the thickness of the deep fascia superficial to the obliquus capitis inferior, and markedly lowered SWVmax and SWVmean in the semispinalis capitis. Together with reduced headache scores and attack frequency, these findings confirm that magnesium sulfate relieves occipital pain by relaxing cervical-muscle spasm in the territory of the greater occipital nerve and thereby enhances the efficacy of pulsed radiofrequency.

Vertebral artery (VA) hypoperfusion is another key contributor to headache. As the main supplier of the posterior circulation, the VA nourishes the brainstem, cerebellum, and occipital cortex. When it is compressed by cervical degenerative changes, uncovertebral joint hypertrophy, or soft-tissue spasm, the lumen first narrows focally. According to Poiseuille’s law and Bernoulli’s principle, flow velocity rises compensatorily at—and just distal to—the stenosis to preserve perfusion (Raza-Taimuri et al., 2026). If compression persists or worsens, overt flow obstruction occurs, downstream perfusion falls, peak systolic velocity (PSV) drops, and notched or even reversed flow may appear, producing ischemia that activates the trigeminovascular system and evokes headache and vertigo (Jellad et al., 2024). During an occipital-neuralgia attack, reflex muscle tension not only aggravates spasm and nerve entrapment but can also compress the VA mechanically, narrowing its lumen, slowing flow, and precipitating or worsening dizziness/vertigo from VA insufficiency (Pareek et al., 2024). Conversely, posterior-circulation ischemia itself produces discomfort that, via neural reflexes, keeps cervical muscles chronically tense, increasing the risk of occipital-nerve compression or inflammation (An et al., 2025). Thus, a vicious cycle is established and represents one of the central pathomechanisms of cervicogenic dizziness with neuralgia. In the present study, Doppler ultrasound was used to compare ipsilateral VA hemodynamics before and after treatment. Flow parameters in the magnesium-sulfate group were significantly better than those in the control group, and this improvement paralleled the superior pain outcomes, confirming that magnesium sulfate, by relieving muscle spasm, also enhances VA perfusion and thereby augments the efficacy of pulsed radiofrequency.

### Limitations

Because the present investigation focused on the additive effect of ultrasound-guided pulsed radiofrequency of the greater occipital nerve combined with perineural magnesium sulfate injection and was conducted at a single center, the sample size was relatively small; larger, multicenter cohort studies are needed for confirmation. Second, pre-operative serum magnesium levels were not measured. To avoid hypermagnesemia-related complications we chose a low dose of 150 mg; previous work has shown this amount to be safe and effective when given intravenously (Olapour et al., 2017) or locally (Ramegowda et al., 2024) to enhance and prolong anesthesia. Future trials should test different dose regimens to establish the optimal safe and effective concentration. Third, the lack of sex-stratified analysis is a limitation of this study. Future research should investigate the potential role of sex hormones in modulating treatment efficacy to enable more personalized therapeutic approaches.

## Conclusion

Perineural magnesium-sulfate injection relieves peri-occipital-nerve muscle spasm and improves vertebral-artery blood flow, thereby potentiating pulsed radiofrequency therapy and markedly reducing headache intensity, duration, and attack frequency while maintaining a high safety and reliability profile.

## Data Availability

The raw data supporting the conclusions of this article will be made available by the authors, without undue reservation.
